# Intraperitoneal clearance as a potential biomarker of cisplatin after intraperitoneal perioperative chemotherapy: a population pharmacokinetic study

**DOI:** 10.1038/bjc.2011.557

**Published:** 2011-12-15

**Authors:** B Royer, E Kalbacher, S Onteniente, V Jullien, D Montange, S Piedoux, A Thiery-Vuillemin, D Delroeux, S Pili-Floury, E Guardiola, M Combe, P Muret, V Nerich, B Heyd, B Chauffert, J-P Kantelip, X Pivot

**Affiliations:** 1 CHU Besançon, Department of Pharmacology, Laboratoire de Pharmacologie Clinique – CHU Jean Minjoz – bvd Fleming, Besançon Cedex F25030, France; 2 Inserm, UMR645, Besançon, France; 3 CHU Besançon, Clinical Investigation Center CIC-BT 506, Besançon, France; 4 Franche-Comté University, IFR133, Besançon, France; 5 CHU Besançon, Department of Oncology, Besançon, France; 6 Department of Pharmacology, Paris Descartes University, Cochin-Saint-Vincent de Paul Hospital, Inserm, U663, Paris, France; 7 CHU Besançon, Department of Surgical Oncology, Besançon, France; 8 CHU Besançon, Department of Anesthesiology and Intensive Care Medicine, Besançon, France; 9 CHU Besançon, Department of Pharmacy, Besançon, France; 10 CHU Amiens, Department of Oncology, Amiens, France

**Keywords:** biomarker, cisplatin, epinephrine, intraperitoneal perioperative chemotherapy, population pharmacokinetics

## Abstract

**Background:**

Intraperitoneal (IP) perioperative chemotherapy with cisplatin is an interesting option in ovarian cancer treatment. A combination of cisplatin with IP epinephrine (already shown to improve IP and decrease systemic platinum (Pt) exposure) was evaluated using a population pharmacokinetic analysis.

**Methods:**

Data from 55 patients treated with cisplatin-based IP perioperative chemotherapy with (*n*=26) or without (*n*=29) epinephrine were analysed using NONMEM.

**Results:**

Epinephrine halves clearance between peritoneum and serum (IPCL) and increases the Pt central volume of distribution, IP exposure and penetration in tissue. IPCL has a better predictive value than any other parameter with respect to renal toxicity.

**Conclusion:**

This confirms that IPCL could be useful in assessing renal toxicity. As IPCL is also linked to tissue penetration and IP exposure, it may be proposed as biomarker. In addition to a Bayesian estimation, we propose a single-sample calculation-way to assess it. Prospective studies are needed to validate IPCL as a biomarker in this context.

Ovarian cancer is the main gynaecologic cause of death in Western countries, with >75% of diagnosed cases presenting with regional or metastatic disease and a 5-year overall survival rate of approximately 30% (Goodman *et al*, 2003). The American Cancer Society estimated that 21 550 new cases of ovarian cancer were diagnosed and 14 600 women died of the disease in 2009 in the United States (Jemal *et al*, 2009).

The standard treatment for advanced ovarian cancer combines optimal cytoreductive surgery (CRS), with intravenous carboplatin–paclitaxel chemotherapy (Aebi and Castiglione, 2008). However, intraperitoneal (IP) chemotherapy (IPC) may be proposed as an additional strategy aimed at achieving a high and more effective cytotoxic local concentration, while decreasing serum concentration (Markman and Walker, 2006). Three randomised phase-III clinical trials show a significant overall and/or progression-free survival advantage when IPC was used after CRS compared with standard doses of intravenous chemotherapy (Alberts *et al*, 1996; Markman *et al*, 2001; Armstrong *et al*, 2006).

The difficulty of the drug to penetrate into the tumour and toxicities linked to high systemic concentrations impede the use of IPC as a ‘routine’ technique (Dedrick and Flessner, 1997; Rowan, 2009). Co-administration of epinephrine and cisplatin (CDDP) was proposed to solve these problems. Indeed, IP administration of this potent vasoconstrictor increased the penetration of platinum (Pt) derivatives into tumours (Duvillard *et al*, 1999; Favoulet *et al*, 2001; Chauffert *et al*, 2003). Such interesting properties led to phase-I studies to assess the feasibility of the IP epinephrine–cisplatin combination in patients with advanced peritoneal carcinomatosis (Molucon-Chabrot *et al*, 2006; Guardiola *et al*, 2010). In particular, Guardiola *et al* (2010) showed that IP epinephrine decreases Pt concentrations in serum and is accompanied by a dramatic reduction in renal toxicity. The purpose of this study was to develop a population pharmacokinetic (POP PK) model of CDDP after perioperative IP administration with epinephrine aiming to assess its impact on the PK parameters and look at the phenomena occurring during this chemotherapy from a different angle. As the addition of IP epinephrine also reduces the rate of renal toxicity, a potential link between PK parameters and the clinical adverse effect of this drug should be investigated.

## Materials and Methods

### Clinical studies and patients

The clinical and PK data used in the analysis were obtained from 55 patients treated with perioperative IPC (PIPC) with (*n*=26) or without (*n*=29) epinephrine (Guardiola *et al*, 2009, 2010). Eligible criterion was recurrent epithelial ovarian cancer, with progression at least 6 months after first-line i.v. chemotherapy based on Pt-containing regimen. Inclusion criteria included: histologically documented recurrent epithelial ovarian cancer confined to the peritoneal cavity (no extra-peritoneal disease), possibility of an optimal CRS aiming to remove all tumour nodules, age over 18, WHO performance status 0 or 1, life expectancy ⩾3 months, and normal haematological, renal and hepatic functions. Owing to the anticipated cardiovascular effects of epinephrine, patients with a history of cardiac pathology were excluded. The study was conducted in compliance with the Declaration of Helsinki and signed informed consent forms were obtained from all patients.

### Treatments

The treatment scheme included 4–8 cycles of i.v. induction chemotherapy with paclitaxel (175 mg m^–2^) and carboplatin (AUC 5), followed by optimal CRS during which PIPC with CDDP alone or the CDDP–epinephrine combination was administered. Perioperative IPC was administered as previously described (Royer *et al*, 2009; Guardiola *et al*, 2010). Epinephrine was then administered at 1 (*n*=11), 2 (*n*=12) and 3 (*n*=3) mg l^–1^ doses. The CDDP-containing baths lasted 1 h, but for some patients (*n*=11) treated with epinephrine, this duration was shortened by 15 min. Indeed, after evaluating the IP Pt concentration, and as these concentrations were low and below the desired threshold (Royer *et al*, 2008), this decision (among others) was taken in an attempt to make this lengthy surgical procedure shorter. Three litres of normal saline, 2.2 Mm Ca^2+^ glucuronate, 1 g l^–1^ Mg^2+^, 2 g l^–1^ KCl and 3 g l^–1^ NaCl were concomitantly intravenously administered for renal toxicity prevention.

### Pharmacokinetic study

Peritoneal and blood sampling for PK analysis was as follows: peritoneal and blood samples were taken 1, 30 and 59 min after the beginning of each of the two 1-h baths (5, 25 and 44 min for the 45-min baths). Additional blood samples were collected 4, 6, 8, 16 and 24 h after the PIPC. The samples obtained 16 h after the beginning of chemotherapy were discarded for the last 11 patients because they were inconvenient and not informative (sampling time around 0300 hours). Blood and peritoneal samples were immediately centrifuged and separated in total and ultrafiltered (Uf) fractions, then frozen until analysis using a validated method based on flameless atomic absorption spectrophotometry using a Varian SpectrAA 220Z graphite furnace spectrometer with Zeeman effect (Varian, Mulgrave, Australia).

### Population pharmacokinetic analysis

Concentration-time data were analysed using the non-linear mixed-effects approach with the NONMEM program version VI.2 software, with double precision (ICON Development Solutions, Elliott City, MD, USA) (Beal *et al*, 1989–2006). The first-order conditional method with the INTERACTION option was used. Both Uf and protein-bound Pt (Pt_B_ = total Pt – Uf Pt) were modelled simultaneously using the following (for details, see Supplementary data S1):



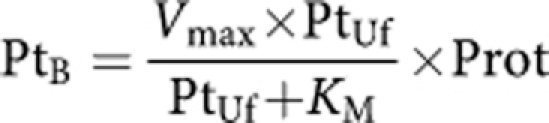



**Figure 1:**
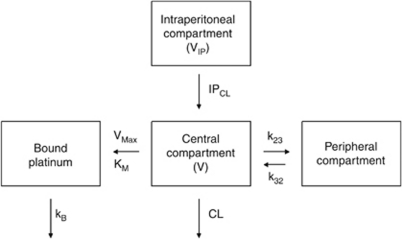
Scheme of the compartmental final model used for the modelling of both Uf and bound Pt. Administration of PIPC was performed in the IP compartment and Uf Pt was transferred to the central (serum) compartment following IP clearance IPCL. Ultrafiltered Pt can change between the central and peripheral compartments and be eliminated following central clearance (CL). Ultrafiltered Pt can also bind to protein following a Michealis–Menten model (*K*_max_, *V*_M_) and thereafter be eliminated (*k*_B_).

This formula was used to model Pt_B_ following the model of Urien and Lokiec (2004). The Pt_B_ was modelled as an additional compartment and underwent first-order elimination from this compartment (Figure 1). This model was only applied to the serum concentrations because IP Pt binding was shown to be very low (Royer *et al*, 2005, 2008).

Identification of the best structural PK model was based on the objective function value and on the inspection of diagnostic graphs using the Perl speaks NONMEM (PsN) (Lindbom *et al*, 2005) and Xpose4 (Jonsson and Karlsson, 1999) toolkits. These programmes were also used to compute the extent of shrinkage in empirical Bayes estimates (eta-shrinkage) and individual predictions (epsilon-shrinkage). Computations were performed in the supercomputer facilities of the Mésocentre de calcul de Franche Comté.

Interindividual variability (IIV) was modelled exponentially. Several error models (i.e., additive, exponential or the combination of both error models) were investigated to describe the residual error. The covariates tested were age, actual body weight, height, body surface area (BSA) calculated according to the Du Bois and Du Bois formula (Du Bois and Du Bois, 1916), body mass index, serum creatinine, creatinine clearance (calculated according to the Cockroft–Gault equation (Cockroft and Gault, 1976)), IP total protein concentration (PRIP), serum total protein concentration (PROT), and presence of epinephrine (EPI – dichotomously coded because Pt plasma concentrations were similar regardless of the dose used (Guardiola *et al*, 2010)). Only covariates with a biologically plausible effect were tested. A covariate was retained in the population model if it produced a decrease in the objective function value of at least 3.84 points compared with the structural PK model, led to a reduction in the IIV of the associated PK parameter, and if a minimum increase in 7 was observed after its removal from the final model.

### Model evaluation

The accuracy and robustness of the final population models were assessed by a (nonparametric) bootstrap method (Green and Duffull, 2003), visual predictive check and normalised prediction distribution errors (Brendel *et al*, 2006; Comets *et al*, 2008).

### Assessment of the epinephrine effect on Pt transfer

To evaluate to what extent epinephrine reduces the amount of Pt transferring from peritoneum to bloodstream, we assessed such an individual transfer rate (RT) as follows (for details, see Supplementary data S1):



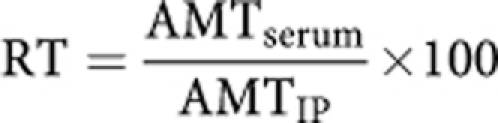



(RT, percentage of Pt, which passed from the peritoneum cavity to the bloodstream, AMT_serum_, amount of Pt in serum, AMT_IP_, amount of IP Pt).

### Pt penetration distance assessment

An individual assessment of the distance at which the interstitial Pt concentration is 5% of that of the IP interface (named 3*x*_0_), that is, an estimation of the Pt penetration, was determined following the studies of Dedrick and Flessner (1997) (for details, see Supplementary data S1):



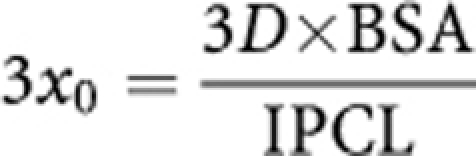



With 3*x*_0_ (*μ*m), BSA (*μ*m^2^), *D* diffusivity (*μ*m^2^ min^–1^) (obtained from El-Kareh and Secomb (2004)) and IPCL is the individual clearance obtained with the Uf model (*μ*m^3^ min^–1^).

### Length of IP Pt exposure

The time during which the IP concentration was over 10 mg l^–1^ was calculated as follows using an equation derived from Kuti *et al* (2004):



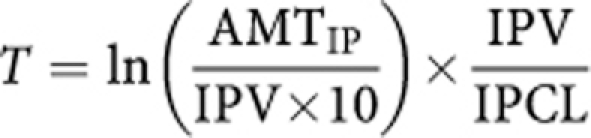



(*T*, time at which the 10 mg l^–1^ concentration is reached in the peritoneum, AMT_IP_ and IPCL as previously described, IPV, individual volume of distribution associated with the IP compartment).

### Assessment of predictive value of PK parameters regarding renal toxicity

As postoperative renal toxicity remains the main adverse effect of PIPC (Guardiola *et al*, 2009; Pili-Floury *et al*, 2011), and as epinephrine dramatically reduces the occurrence of such toxicity, we assessed a potential link between several PK parameters (IPCL, CL, AUC_serum_ and AUC_IP_) and this toxicity. Renal toxicity was assessed according to RIFLE classification based on postoperative serum creatinine level changes from baseline (for details, see Supplementary data S1). Receiver operating characteristics curves were then calculated for each PK parameter. This allowed us to determine a threshold that was used to assess sensitivity, specificity, positive predictive value, negative predictive value and the odds ratio for each PK parameter.

## Results

### Patient population and structural PK model

Clinical data of the patient population are summarised in Table 1. A total of 316 IP samples and 577 Uf plasma and 577 bound samples were used for the analysis. A three-compartment model with first-order elimination from the serum (central) compartment best fitted the data of all patients (Figure 1). The corresponding PK parameters were IPCL, volume of distribution associated with the IP compartment (IPV), clearance from the serum (central) compartment (CL), volume of distribution associated with the serum central compartment (V), Michaelis–Menten constants used to model covalent binding to protein (*V*_max_ and *K*_M_), elimination constant rate of Pt_B_ (*k*_B_), and the rate constants between serum central and peripheral compartments (*k*_23_ and *k*_32_) (Table 2). Interindividual variability on *k*_23_, *k*_32_, *K*_M_ and *k*_B_ could not be estimated. A correlation between V and *V*_max_ was observed and estimated. The error model includes both proportional and additive models, but the latter was only applied to IP concentrations.

### Covariates

Only epinephrine led to a significant decrease in IIV. Epinephrine decreased the objective functional value and IIV for both IPCL and V. The administration of epinephrine led to an IPCL decrease in 53.1% and an increase in V of 80.5%. Associated variability was reduced by 48.9 and 53.4%.

### Model evaluation

**Figure 2:**
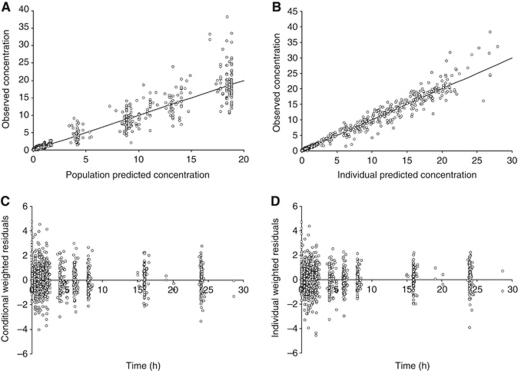
Scatterplots allowing us to assess the goodness-of-fit for the final models. Graphs represent model-Predicted (PRED) (**A**) and Individual Predicted (**B** – shrinkage ⩽11.0%) concentrations plotted *vs* Observed concentrations, as well as Conditional (**C**) and Individual (**D**) Weighted Residuals *vs* Time. These concentrations were observed for the concentrations obtained IP, in serum and for bound Pt. The scatterplots for each compartment are displayed in Supplementary Figure S2 of the Supplementary data.

The goodness-of-fit plots for all samples of the final model are shown in Figures 2A–D. The goodness was confirmed for each studied compartment (Supplementary Figures S2 – supplementary material). The bootstrapped mean and 95CI of the parameter estimates are summarised in Table 2.

The figures corresponding to the posterior visual predictive check and the normalised prediction distribution errors evaluation confirm the satisfactory predictability of the final population PK models (Supplementary material – Supplementary Figures S3A and S3B, respectively). In particular, the 15-min reduction of PIPC was correctly modelled (see Supplementary Figure S3A) and thus enables us to analyse these patients together with those treated with the 1-h baths.

### Impact of epinephrine effect on Pt

**Figure 3:**
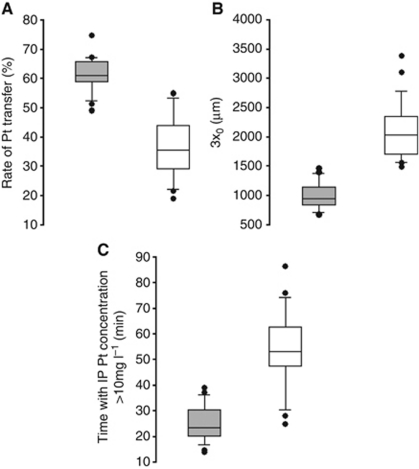
Effect of epinephrine on Pt behaviour during PIPC. (**A**) The effect of epinephrine on individual rate of transfer of Uf Pt from peritoneum to bloodstream. (**B**) Assessment of the effect of epinephrine on the individual Pt penetration. 3*x*_0_ (*μ*m) is the distance in peritoneal tissue at which the concentration difference between tissue and blood perfusing this tissue decreased to 5% of its maximal value. (**C**) Time during which the IP Pt concentration is over 10 mg l^–1^. The data were obtained for patients treated with cisplatin with (white box) or without (grey box) epinephrine. ‡ means *P*<10^−4^ (*t*-test) as compared with values observed without epinephrine.

The decrease in IPCL because of epinephrine reduced the rate of transfer of Pt from peritoneum to bloodstream by 40.2% (mean individual, *P*<10^−4^) (Figure 3A). This was accompanied by an increase in the length of time during which IP Pt concentration was higher than 10 mg l^–1^ (a concentration associated with the cytotoxicity of a resistant cell line (Royer *et al*, 2005; Facy *et al*, 2011)). After epinephrine administration, this duration more than doubled (25.1±6.8 min *vs* 53.9±13.5 min, *P*<10^−4^) (Figure 3C). Interestingly, the addition of epinephrine in the peritoneal bath also led to an increase in the calculated Pt penetration in interstitial tissue. The mean Pt penetration was 992±219 *μ*m without epinephrine *vs* 2100±473 *μ*m with epinephrine (*P*<10^−4^) (Figure 3B).

### Selection of IPCL as the best marker of toxicity

Of the 29 patients who did not receive epinephrine, 28 were able to undergo renal toxicity assessment (Supplementary data S1). Of these, 14 underwent high clinical toxicity (IF) while this toxicity was lower in the 14 other patients. The 26 patients treated with epinephrine did not develop renal injury or failure.

Receiver operating characteristics curves indicate that IPCL, AUC_IP_ and AUC_serum_, but not CL are able to discriminate patients with a potential risk of renal toxicity (Supplementary data – Supplementary Figure S4). For these three PK parameters, predictive values (sensitivity, specificity, positive predictive value, negative predictive value and odds ratio) show that IPCL seems to be the best parameter for predicting potential CDDP toxicity (Table 3). To reduce the number of samples and simplify the sampling schedule, the Bayesian estimation of IPCL was assessed with only the last IP sample of each bath. These estimations led to satisfactory values of IPCL with a higher positive predictive value than IPCL obtained with all samples, but a lower negative predictive value (Table 3 and Supplementary data – Supplementary Figure S4). However, a biomarker must be easily accessible in order to be effective. We therefore aimed to calculate IPCL directly using the Uf IP concentration of Pt with the following formula:



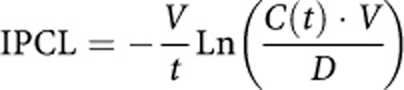



(*V* (l), volume in which Pt is administered with the dose *D* (mg); *C*(*t*), concentration of Uf IP Pt measured at the time *t*).

Taking the last values of Pt of each bath, the calculated IPCL values give a lower AUC for receiver operating characteristics curve (Supplementary data – Supplementary Figure S4), but provide good predictive values that are similar to those observed with the Bayesian estimation or with all the samples (Table 3).

## Discussion

Population pharmacokinetic studies give another view of the pharmacokinetic phenomena taking place during this chemotherapy. We described the effect of epinephrine on V and on the clearance between IP and serum. The considerable increase in V after epinephrine administration was somewhat surprising. The role of epinephrine in the increase in V may be explained by a *β*1-adrenergic-mediated myocardial stimulation (positive inotropic and chronotropic actions) and a *β*2-mediated peripheral vasodilatation (decrease in peripheral resistance). Consequently, the blood flow distribution in tissues increases together with the rate and extent of Pt transfer outside the capillaries, resulting in lower total Pt serum concentration (Fagiolino *et al*, 2006). Of note, an increase in V was also observed when epinephrine was used in combination with local anaesthetics after perineural administration (Tucker and Mather, 1979) meaning that this effect is probably not related to the mode of administration, nor to the combined drug. The decrease in IPCL is thought to be partly due to a reduction in splanchnic blood flow (*α*1-adrenergic vasoconstriction of the peritoneal vessels). The combination of the increase in V and the decrease in IPCL may explain the decrease in concentrations observed after epinephrine administration (Guardiola *et al*, 2010).

Interestingly, the POP PK study provides access to individual IPCL. This enabled us to assess the individual Pt penetration in peritoneal tissue. The effect of epinephrine was clear-cut (Figure 3): the mean Pt penetration more than doubled. Although these values of penetration were obtained with a theoretical model, they were in the same range as those observed in animal models (Los *et al*, 1989, 1990; Duvillard *et al*, 1999; Favoulet *et al*, 2001; Chauffert *et al*, 2003; Esquis *et al*, 2006) and those obtained after hyperthermia in humans (van de Vaart *et al*, 1998). However, in animal models, epinephrine was shown to be more effective than hyperthermia in enhancing intratumoural concentration of Pt while decreasing its peripheral concentration and extra-peritoneal tissue penetration (Facy *et al*, 2011). Moreover, epinephrine increases the time during which the concentration is above 10 mg l^–1^. These effects are interesting as, to be clinically relevant, the high and sustained IP Pt level must result in significant Pt accumulation in tumour nodules (Rao *et al*, 2007; Van der Speeten *et al*, 2009).

The administration of epinephrine led to the suppression of the clinically relevant renal toxicity previously observed in around half of the patients (Supplementary data S1). As we observed that epinephrine administration led to a great difference in both creatinine ratio and serum Pt concentrations (Guardiola *et al*, 2010), we assumed that, thanks to the POP PK study providing access to individual PK parameters, this could help us to determine a PK parameter linked to renal toxicity. IPCL was the best PK parameter for predicting renal toxicity, showing the best predictive values. This is unsurprising, as this parameter pharmacokinetically drives both the IP and serum AUC. Interestingly, IPCL may also be used to compute pharmacodynamic parameters. However, as we could not directly link this parameter to efficacy, further prospective studies are needed to correlate this parameter with efficacy. This parameter could thus be used to assess both toxicity and possibly efficacy of cisplatin perioperative IP administration. Indeed, there are a few biomarkers in the field of IPC. Although CA125 was proposed as a predictor of progression-free and overall survival in ovarian cancer patients before IPC (Juretzka *et al*, 2007), its interest is controversial (Juretzka *et al*, 2007; Richardson *et al*, 2008; Richard *et al*, 2010). Moreover, as there is neither a marker of toxicity nor of efficacy for cisplatin-based IPC, IPCL could be useful in this context. However, further studies, with more homogeneous patients than those we studied, are needed to assess this parameter prospectively in terms of toxicity and efficacy. This is the condition required to consider IPCL as a biomarker of cisplatin when after PIPC.

A biomarker needs to be easily assessable in biological fluids of patients. However, in this study, IPCL was obtained after an intensive sampling schedule and a POP PK analysis, making access to this parameter difficult. We first aimed to reduce the number of samples to the last IP sample of each bath. Using the final model, the Bayesian estimation of IPCL with only these two samples was satisfactory. Second, to make the IPCL assessment possible without the POP PK approach, we attempted to calculate it considering the following approximations: V was set to the volume of chemotherapy. This approximation was possible because this parameter displays low variability when estimated with POP PK (Royer *et al*, 2009; Cotte *et al*, 2011), and both low IP protein concentration and low protein binding leads to this volume being close to V. Thus, considering both the administered dose and volume (easily available with an open procedure), the IP Uf concentration obtained just before the end of IPC can be used to calculate the IPCL directly using the equation previously described. Using this method, we obtained IPCL predictive values similar to those obtained using NONMEM (with the full model or the Bayesian estimation), which makes this parameter easily available even without POP PK modelling (with or without epinephrine). However, the approximations used to calculate this parameter may weaken its predictive value. For instance, the individual estimation of IPCL using the POP PK approach indirectly takes into account parameters that dictate the Pt transfer, such as the permeability and the effective contact area (Supplementary data S1). Direct calculation of IPCL with the proposed formula does not. In the event of huge preoperative malignant ascites, POP PK estimation of IPCL may be more realistic than the calculated approach. The estimation of the surface of the contact area by BSA for the interstitial penetration assessment may also be biased. For these reasons, it seems very important to assess the predictive values of these parameters in prospective studies in which these potential biases should be detected and evaluated.

In conclusion, the present POP PK analysis aimed to propose a potential biomarker of cisplatin after PIPC. Two characteristics of this study make this possible. First, the POP PK approach provides access to individual PK parameters. Second, the administration of epinephrine led to a dramatic reduction in renal toxicity. Taken together, these approaches led to a correlation study, which showed that the IPCL appears the best parameter linked to toxicity, and that this parameter could potentially be related to efficacy. Given that the POP PK approach is not widely available, we propose a more simple approach to assess this parameter. Although the assessment of IPCL with only one sample using the proposed equation is not as precise as the Bayesian estimation, this approach may be universally adopted with a view to a prospective study to confirm this approach and determine an essential threshold for patient follow-up.

## Figures and Tables

**Table 1:** Summary of baseline characteristics of patients who underwent IP peroperative chemotherapy with CDDP combined or not with epinephrine

	IP CDDP (*n*=29)	IP CDDP–epinephrine association (*n*=26)	All patients (*n*=55)
Age (years)	58.2 (25.5–75.0)	59.1 (26.6–70.8)	58.3 (25.5–75.0)
Actual weight (kg)	57 (49–84)	61 (49–85)	60.5 (49–85)
Lean body mass (kg)	43.0 (38.4–56.2)	43.8 (36.6–51.7)	43.6 (36.6–56.2)
Height (cm)	163 (150–176)	160 (150–178)	161.5 (150–178)
Body mass index (kg m^–2^)	23.6 (17.9–28.5)	23.4 (20.2–31.2)	23.4 (17.9–31.2)
Body surface area (m^2^)^a^	1.26 (1.48–2.01)	1.63 (1.42–1.92)	1.63 (1.42–2.01)
Lean body mass (kg)^b^	44.7 (38.4–56.2)	44.5 (36.6–51.7)	44.7 (36.6–56.2)
Serum creatinine (μmol l^–1^)	57.1 (32–79)	62.5 (48–88)	58.0 (32–88)
Creatinine clearance (ml min^–1^)^c^	97.5 (72.6–182.8)	80.0 (58.2–133.2)	94.0 (58.2–182.8)
Total protein concentration in serum (g l^–1^)	31.0 (16–42)	36.5 (22–56)	34.0 (16–56)

Abbreviations: CDDP=cisplatin; IP=intraperitoneal.

a Calculated according to the Dubois and Dubois formula.

b Calculated according to the James formula.

c Estimated with the Cockcroft and Gault formula.

Data are presented as median (range).

**Table 2:** Population pharmacokinetics parameters of Pt estimated from the final model and bootstrap validation (500 resamplings)

		Bootstrap analysis		
Parameter	Original data set estimate (%RSE)	Mean	95% CI	Shrinkage (%)
Structural model				
IPV (l)	3.10 (3.1)	3.11	2.94 to 3.26	
				
*IPCL (l h*^*–1*^*)= θ*_*1*_*+ θ*_*2*_ *x EPI*				
θ_1_	4.66 (4.3)	4.66	4.34 to 5.02	
θ_2_	–0.531 (6.9)	–0.533	–0.471 to –0.586	
CL (l h^–1^)	9.63 (5.2)	9.61	8.77 to 10.49	
				
*V (l) = θ*_*3*_*+ θ*_*4*_ *x EPI*				
θ_3_	21.4 (5.9)	21.40	19.40 to 24.49	
θ_4_	0.805 (26.3)	0.778	0.472 to 1.188	
*k*_23_ (h^−1^)	0.632 (3.7)	0.635	0.595 to 0.678	
*k*_32_ (h^−1^)	0.0425 (4.4)	0.0424	0.0395 to 0.0450	
*V*_max_ (mg l^–1^)	0.0123 (9.2)	0.0124	0.0106 to 0.0142	
*K*_M_ (mg l^–1^)	2.00 (10.1)	1.98	1.66 to 2.37	
*k*_B_ (l h^–1^)	0.382 (7.2)	0.381	0.330 to 0.437	
				
*Interindividual variability*				
IIV_IPV_ (%CV)	19.7 (25.9)	19.1	14.9 to 23.2	9.7
IIV_IPCL_ (%CV)	22.3 (19.0)	21.9	17.9 to 25.3	11.0
IIV_CL_(%CV)	39.4 (28.2)	38.0	30.1 to 47.3	9.1
IIV_V_ (%CV)	26.4 (42.5)	26.5	17.0 to 38.1	6.8
IIV_Bmax_ (%CV)	51.3 (12.2)	50.8	44.6 to 56.9	0.7
ρ *V*/*V*_max_	–0.124 (29.3)	–0.123	–0.062 to –0.197	
				
*Residual errors*				
ε_1_ (%CV)	17.8 (9.6)	17.7	16.4 to 19.2	7.3
ε_2_ (mg l^–1^)	0.098 (7.0)	0.098	0.083 to 0.114	

Abbreviations: θ=value of the parameter associated with the equation of the covariate; ε_1_=exponential part of the residual error; ε_2=_additive part of the residual error; CI=confidence interval; %CV=percentage of coefficient of variation; CL=clearance associated with the serum (central) compartment; IPCL=IP clearance; IIV=interindividual variability; Pt=platinum; %RSE=relative standard error; V and IPV=volume of distribution associated with the serum central and IP compartments; *V*_max_ and KM=the Michaelis–Menten constants used to model covalent binding to protein; *k*_B_=the elimination constant rate of Pt-bound Pt to protein; *k*_23_, *k*_32_=rate constants between central and peripheral compartments.

**Table 3:** Predictive values of different PK parameters with respect to renal toxicities

	AUC of ROC curve	Threshold	Sensitivity	Specificity	Positive predictive value	Negative predictive value	OR (95% CI)
IPCL	0.909	4.1	0.929	0.825	0.650	0.971	61.3 (9.5–395.0)
CL	0.514		NA	NA	NA	NA	NA
AUC_IP_	0.855	19.6	0.714	0.800	0.556	0.889	10.0 (2.6–38.1)
AUC_serum_	0.854	4.5	0.857	0.725	0.522	0.935	15.8 (3.5–72.3)
IPCL_Bayes_	0.923	4.5	0.786	0.872	0.688	0.919	24.9 (5.6–111.4)
IPCL_calc_	0.892	3.2	0.857	0.821	0.632	0.941	27.4 (5.7–132.2)

Abbreviations: AUC_IP_=area under the concentration curve observed in the peritoneal compartment; AUC_SERUM_=area under the concentration curve observed in the central compartment; CI=confidence interval; CL=clearance from the serum (central) compartment; IP=intraperitoneal; IPCL=IP clearance; IPCL_Bayes_=IPCL estimated with two IP samples obtained at the end of each bath and assessed with a Bayesian estimation, IPCL_calc_=IPCL estimated with two IP samples obtained at the end of each bath and assessed with the formula described in the paper; NA=not applicable; OR=odds ratio. The thresholds were determined after receiver operating characteristics (ROC) curve assessment and their units are l h^–1^ for IPCL and mg h l^–1^ for both AUCs. Predictive values of CL were not evaluated because of too bad ROC evaluation.
