# Supplementary material for: Intraperitoneal clearance as a potential biomarker of cisplatin after intraperitoneal perioperative chemotherapy: a population pharmacokinetic study
Source: Br J Cancer. 2011 Dec 15;106(3):460–7. doi: 10.1038/bjc.2011.557 (PMC3273361; doi:10.1038/bjc.2011.557)
Supplement: Supplementary data S1 [file bjc2011557x1.doc]

**Modeling of protein-bound platinum**

Both Uf and protein-bound Pt were modeled simultaneously. Firstly, bound Pt (Pt_B_ = Total Pt minus Uf Pt) was modeled using *in vitro* data (with different protein concentrations, obtained from Royer *et al* [1]) based on the saturable Michaelis-Menten covalent binding model [2] as follows:

$$Pt_{B}=\frac{V_{\text{max}}\times Pt_{Uf}}{Pt_{Uf}+K_{M}}$$

(Vmax and K_M_, Michaelis-Menten constants used to model covalent binding to protein).

A better fitting was obtained when protein concentration (Prot) was added as follows (data not shown):

$$Pt_{B}=\frac{V_{\text{max}}\times Pt_{Uf}}{Pt_{Uf}+K_{M}}\times\text{Pr}ot$$

**Assessment of the epinephrine effect on Pt transfer**

To evaluate to what extent epinephrine reduces the amount of Pt transferring from peritoneum to bloodstream, we assessed such an individual transfer rate. The amount of Pt in each compartment for 24 hours was calculated using the formula

$$AMT=CL\times AUC$$

(AMT: amount of Pt in the compartment considered, CL: clearance from the compartment considered, AUC: area under the concentration curve in the compartment considered). The individual clearances were obtained from the NONMEM results and the AUC were calculated as previously described [3]. Briefly, a supplementary compartment was added to the model for both IP and serum. In these compartments, the amount of Pt divided by the corresponding volume of distribution was implemented, aggregating the concentration measurements over time. The AUC was determined by the value obtained in these compartments at the time of the last sample. The individual rate of transfer was calculated by

$$RT=\frac{AMT_{serum}}{AMT_{IP}}\times100$$

(RT: percentage of Pt which passed from the peritoneum cavity to the bloodstream, AMT_serum_: amount of Pt in serum, AMT_IP_: amount of IP Pt).

**Platin penetration distance assessment**

Dedrick and Flessner described the evolution of the free drug concentration in the interstitial tissue of the peritoneal wall depending on both peritoneal and blood perfusing the tissue concentrations and on the distance from the serosal surface [4]. They also described a characteristic diffusion length, named x_0_, at which the concentration difference between tissue and blood decreased to 37% of its maximal value (peritoneal concentration). They also considered that 3x_0_, the distance from serosal surface at which this difference is 5% of the maximal value, could be a useful parameter to assess and compare drug penetration in tumors. x_0_ can be evaluated as follows:

$$\text{x}_{0}=\sqrt{\frac{\text{D}}{\text{k}}}$$

where x_0_ is the tissue distance from the serosal surface (µm), D is the diffusivity (µm^2^/min) and k the rate constant of drug removal from the tissue. Similarly, these authors also showed that the tissue permeability (P) can be calculated as follows:

$$\text{P}=\sqrt{\text{Dk}}$$

Taken together, these equations enable us to estimate x_0_ from D and P:

$$\text{x}_{0}=\frac{D}{P}$$

Interestingly, Jones *et al* showed that IP clearance (IPCL) gives directly (and is) the peritoneal permeability area (A) product (PA) [5], as defined by Dedrick *et al* [4, 6], which governs the transfer between IP compartment and bloodstream. As the peritoneal surface can be satisfactorily estimated by the BSA [7],

$$IPCL=PA=P\times BSA then \frac{1}{P}=\frac{BSA}{IPCL}$$

Then, the 3x_0_ can be expressed as follows:

$$3x_{0}=\frac{3D\times BSA}{IPCL}$$

With 3x_0_ (µm), BSA (µm^2^), D diffusivity (µm^2^/min) (obtained from El-Kareh and Secomb [8]) and IPCL is the individual clearance obtained with the Uf model (µm^3^/min). This provides an individual assessment of the distance at which the interstitial Pt concentration is 5% of that of the IP interface, that is, an estimation of the Pt penetration.

**Assessment of predictive value of PK parameters regarding renal toxicity**

Renal toxicity was assessed according to RIFLE classification based on postoperative serum creatinine level changes from baseline [9]. Briefly, patients were classified N (No or minimal renal function alteration), R (renal Risk), I (renal Injury), or F (renal Failure), if at least one value of the ratio between postoperative serum creatinine value / baseline serum creatinine value remained ≤ 1.5 or increased above 1.5, 2, or 3, respectively. Patients were also classified F if at least one value of postoperative serum creatinine was > 350 μmol/L or if renal dialysis was required postoperatively. The clinical relevance of alterations in renal functions led to split the patients into two groups: N and R *vs* I and F [10]. The Renal toxicity status of the 54 patients evaluated after IP perioperative chemotherapy with or without epinephrine is detailed in the table below.

|  | N | R | I | F |
| --- | --- | --- | --- | --- |
| Without epinephrine | 10 | 4 | 8 | 6 |
| With epinephrine | 18 | 8 |  |  |

1. Royer B, Guardiola E, Polycarpe E et al. Serum and intraperitoneal pharmacokinetics of cisplatin within intraoperative intraperitoneal chemotherapy: influence of protein binding. Anticancer Drugs 2005; 16: 1009-1016.

2. Obach RS, Kalgutkar AS, Soglia JR, Zhao SX. Can in vitro metabolism-dependent covalent binding data in liver microsomes distinguish hepatotoxic from nonhepatotoxic drugs? An analysis of 18 drugs with consideration of intrinsic clearance and daily dose. Chem Res Toxicol 2008; 21: 1814-1822.

3. Royer B, Jullien V, Guardiola E et al. Population pharmacokinetics and dosing recommendations for cisplatin during intraperitoneal peroperative administration: development of a limited sampling strategy for toxicity risk assessment. Clin Pharmacokinet 2009; 48: 169-180.

4. Dedrick RL, Flessner MF. Pharmacokinetic problems in peritoneal drug administration: tissue penetration and surface exposure. J Natl Cancer Inst 1997; 89: 480-487.

5. Jones RB, Collins JM, Myers CE et al. High-volume intraperitoneal chemotherapy with methotrexate in patients with cancer. Cancer Res 1981; 41: 55-59.

6. Dedrick RL, Myers CE, Bungay PM, DeVita VT, Jr. Pharmacokinetic rationale for peritoneal drug administration in the treatment of ovarian cancer. Cancer Treat Rep 1978; 62: 1-11.

7. Albanese AM, Albanese EF, Mino JH et al. Peritoneal surface area: measurements of 40 structures covered by peritoneum: correlation between total peritoneal surface area and the surface calculated by formulas. Surg Radiol Anat 2009; 31: 369-377.

8. El-Kareh AW, Secomb TW. A theoretical model for intraperitoneal delivery of cisplatin and the effect of hyperthermia on drug penetration distance. Neoplasia 2004; 6: 117-127.

9. Bellomo R, Ronco C, Kellum JA et al. Acute renal failure - definition, outcome measures, animal models, fluid therapy and information technology needs: the Second International Consensus Conference of the Acute Dialysis Quality Initiative (ADQI) Group. Crit Care 2004; 8: R204-212.

10. Pili-Floury S, Royer B, Bartholin F et al. Protective effect of intra-peritoneal epinephrine on postoperative renal function after cisplatin-based intra-peritoneal intra-operative chemotherapy. Eur J Obstet Gynecol Reprod Biol 2011; 156: 199-203.
