# Supplementary material for: Intraperitoneal clearance as a potential biomarker of cisplatin after intraperitoneal perioperative chemotherapy: a population pharmacokinetic study
Source: Br J Cancer. 2011 Dec 15;106(3):460–7. doi: 10.1038/bjc.2011.557 (PMC3273361; doi:10.1038/bjc.2011.557)
Supplement: Supplementary Figure S2 [file bjc2011557x2.doc]

**Figure S2A**: Scatterplots of the final population PK model for Pt concentrations observed in peritoneum compartment. Black line, line of identity.

**Figure S2B**: Scatterplots of the final population PK model for Pt concentrations observed in serum. Black line, line of identity.

**Figure S2C**: Scatterplots of the final population PK model for Pt bound to protein. Black line, line of identity.
