# Supplementary material for: Intraperitoneal clearance as a potential biomarker of cisplatin after intraperitoneal perioperative chemotherapy: a population pharmacokinetic study
Source: Br J Cancer. 2011 Dec 15;106(3):460–7. doi: 10.1038/bjc.2011.557 (PMC3273361; doi:10.1038/bjc.2011.557)
Supplement: Supplementary Figure S3 [file bjc2011557x3.doc]

**Figure S3A**: Visual Predictive Check (VPC) results : Observed Pt plasma concentrations were plotted with median (dotted line) and 5th and 95th percentile (solid lines) of estimated concentrations for the following compartments: IP without (A) and with EPI (B), serum without (C) and with EPI (D) and bound Pt (E). Of note, no break were drawn for the lines of the figure B as there was an overlap of the administration schedules (cf methods). Interestingly, a fall of concentrations around 1 hour, corresponding to the end of the first bath with the first schedule (1-hour administration) is surrounded by high concentrations corresponding to the beginning of the second bath with the second schedule (45-minutes administration). This fall is correctly estimated by the model as objectified by the fall of median and percentile lines.

**Figure S3B**: Scatter plot of npde: A- Quantile-quantile plots (QQ-plots) comparing the distribution of NPDE to the theoretical standard normal [N (0,1)] distribution; B - histogram showing NPDE with the density of N (0,1) overlayed; scatterplots of C - NPDE versus time; and D - NPDE versus predicted concentrations
