# Supplementary material for: Intraperitoneal clearance as a potential biomarker of cisplatin after intraperitoneal perioperative chemotherapy: a population pharmacokinetic study
Source: Br J Cancer. 2011 Dec 15;106(3):460–7. doi: 10.1038/bjc.2011.557 (PMC3273361; doi:10.1038/bjc.2011.557)
Supplement: Supplementary Figure S4 [file bjc2011557x4.doc]

A

B

C

D

**Figure S4**: ROC curves obtained to test the discrimination power of four PK parameters with respect to renal toxicity as previously described (table S4). The PK parameters studied were the IP clearance (IPCL), the central (serum) compartment clearance (CL), the area under the concentration curve in the IP and serum compartments (AUC_IP_ and AUC_serum_ respectively), IIPCLBayes: IPCL estimated with two IP samples obtained at the end of each bath and assessed with a Bayesian estimation, IPCLcalc: IPCL estimated with two IP samples obtained at the end of each bath and assessed with the formula described in the manuscript.

F

E
